# A Translatable Predictor of Human Radiation Exposure

**DOI:** 10.1371/journal.pone.0107897

**Published:** 2014-09-25

**Authors:** Joseph Lucas, Holly K. Dressman, Sunil Suchindran, Mai Nakamura, Nelson J. Chao, Heather Himburg, Kerry Minor, Gary Phillips, Joel Ross, Majid Abedi, Robert Terbrueggen, John P. Chute

**Affiliations:** 1 Information Initiative at Duke, Duke University, Durham, NC, United States of America; 2 Institute for Genome Sciences and Policy, Duke University, Durham, NC, United States of America; 3 Division of Hematologic Malignancies and Cellular Therapy, Department of Medicine, Duke University Medical Center, Durham, NC, United States of America; 4 Department of Immunology, Duke University, Durham, NC, United States of America; 5 Dxterity Diagnostics, Los Angeles, CA, United States of America; 6 Division of Hematology/Oncology, Department of Medicine, Broad Stem Cell Research Center, Jonnson Comprehensive Cancer Center, U.C.L.A., Los Angeles, CA, United States of America; Dresden University of Technology, Germany

## Abstract

Terrorism using radiological dirty bombs or improvised nuclear devices is recognized as a major threat to both public health and national security. In the event of a radiological or nuclear disaster, rapid and accurate biodosimetry of thousands of potentially affected individuals will be essential for effective medical management to occur. Currently, health care providers lack an accurate, high-throughput biodosimetric assay which is suitable for the triage of large numbers of radiation injury victims. Here, we describe the development of a biodosimetric assay based on the analysis of irradiated mice, *ex vivo*-irradiated human peripheral blood (PB) and humans treated with total body irradiation (TBI). Interestingly, a gene expression profile developed via analysis of murine PB radiation response alone was inaccurate in predicting human radiation injury. In contrast, generation of a gene expression profile which incorporated data from *ex vivo* irradiated human PB and human TBI patients yielded an 18-gene radiation classifier which was highly accurate at predicting human radiation status and discriminating medically relevant radiation dose levels in human samples. Although the patient population was relatively small, the accuracy of this classifier in discriminating radiation dose levels in human TBI patients was not substantially confounded by gender, diagnosis or prior exposure to chemotherapy. We have further incorporated genes from this human radiation signature into a rapid and high-throughput chemical ligation-dependent probe amplification assay (CLPA) which was able to discriminate radiation dose levels in a pilot study of *ex vivo* irradiated human blood and samples from human TBI patients. Our results illustrate the potential for translation of a human genetic signature for the diagnosis of human radiation exposure and suggest the basis for further testing of CLPA as a candidate biodosimetric assay.

## Introduction

The possibility of a terrorist-mediated attack using a dirty bomb or an improvised nuclear device is considered a major threat to both national security and public health [1]–[3]. In the past decade, substantial efforts have been made in the federal, state and private sectors to prepare for an effective medical response in the event of a radiation disaster in a U.S. city [4], [5]. As illustrated in the aftermath of the Fukushima power plant accident, the threat of exposure to even a relatively small amount of ionizing radiation can have paralyzing effects on a global scale [6]. In the event of a radiation disaster in which potentially thousands of people might be exposed to life-threatening doses of radiation, it will be critical for caregivers to be able to separate those who have suffered radiation injury from the “worried well.” Currently, methods to assess absorbed radiation dose in people include time to symptoms of nausea and vomiting, lymphocyte depletion kinetics and severity of neutropenia [3], [7]–[11]. Two of the more well-developed algorithms for utilizing such clinical information include the European Medical Treatment Protocols for Radiation Accident (METREPOL) system and the Radiation Event Medical Management (REMM) algorithm developed by the Office of the Assistant Secretary for Preparedness and Response in H.H.S. [10], [11]. While these clinical algorithms can provide indication in some victims as to their radiation status and level of exposure, a high throughput, quantitative assay to predict radiation status and radiation dose levels in humans could be of substantial benefit in the triage of radiation mass casualties [7], [9], [11], [12]. The successful development and validation of such an assay for human radiation injury would address a major gap in the capability of health care providers to assess and properly treat radiation victims.

As a strategy to develop a single, practical assay to accurately assess radiation status following acute radiation exposure, we measured the global gene expression of a highly radiosensitive and easily accessible cell population, peripheral blood (PB) cells, in mice and humans [13]. Our approach leveraged the power of genome-scale assessment of cellular response to radiation, coupled with advanced computational tools, to predict, with a single test, the radiation status of an individual and also to discriminate radiation dose levels among victims [13]. In our initial studies of C57Bl6 mice, we demonstrated that gene expression profiles from the peripheral blood of irradiated mice were able to predict the radiation status of non-irradiated and irradiated mice with 100% accuracy and also could discriminate mice exposed to 50 cGy, 200 cGy or 1000 cGy from each other [13]. In independent studies of healthy adult people and patients treated with total body irradiation (TBI) as conditioning for hematopoietic cell transplantation, we showed that a human gene expression profile of radiation exposure was able to predict human radiation status with approximately 90% accuracy and that such signatures were not confounded by variables such as time from exposure, genotype or bacterial infection [13], [14]. We have also shown that gene expression profiles developed in mice irradiated with partial-body exposure were capable of predicting the radiation status of animals exposed to heterogeneous exposures [15]. These results provided proof of principle that a PB assay for radiation status utilizing gene expression profiles could be applied as a single, practical assay for radiation triage in the event of a radiation mass casualty scenario. Here, we sought to develop a highly refined classifier of human radiation exposure via the combined analysis of irradiated mice, *ex vivo*-irradiated human PB cells and human TBI patients. Our studies reveal that gene expression profiles of radiation injury developed in mice models alone, without the aid of human model systems, are not accurate in predicting human radiation status. In contrast, a gene expression classifier generated from analysis of human ex vivo irradiated PB and human TBI patients was able to accurately predict radiation dose levels in all human samples tested. Furthermore, we have translated this refined signature of human radiation exposure to a diagnostic platform, CLPA, and preliminary results suggest this platform has high accuracy in predicting human radiation status.

## Materials and Methods

### Ethics Statements

All research involving human participants was approved by the Duke University Institutional Review Board.

### Murine Irradiation

Ten to 12 week old female and male C57Bl6 mice (Jackson Laboratory, Bar Harbor, ME) were housed in the Duke Cancer Center Isolation Facility and all protocols were approved by the Duke University Animal Care and Use Committee (Protocol Number A037-10-02). Mice were exposed to 0, 100, 200, 300, 450, 600, 800 or 1050 cGy total body irradiation (TBI) via a Cs137 irradiator at a dose rate of 480 cGy/min. Peripheral blood samples were collected via terminal cardiac puncture at 6, 24, 48 and 72 hrs, as well as at day +5 and +7 following TBI.

### Murine LPS and GCSF Exposure

Ten to 12 week old female and male C57Bl6 mice were treated subcutaneously with 100 µg of lipopolysaccharide (LPS) endotoxin from *E. coli* 055:B5 (Sigma-Aldrich, St. Louis, MO), 100 µg/kg granulocyte-colony stimulating factor (GCSF, Murine recombinant GCSF or vehicle (0.1% FBS in PBS) at 2 hours following 0 cGy, 200 cGy, 300 cGy or 600 cGy TBI. PB was collected at 6 and 24 hours following TBI.

### Human *Ex Vivo* Irradiation

One tube from each healthy, consented adult donor was randomly assigned to a radiation group and labeled with the target dose. Tubes were irradiated according to their designated doses of 0 cGy, 150 cGy, 300 cGy, or 600 cGy. Radiation exposure times from the Cs137 irradiator were calculated to achieve the target doses specific for tubes filled with blood and inserted in the rotating test tube holder. The dose rate was 480 cGy/min for the duration of the study.

### Human Total Body Irradiation (TBI)

Adult patients (n = 45, 22 females, 23 males), ages 21 to 66, were evaluated at the Duke University Adult Bone Marrow Transplantation Program and enrolled in a Duke IRB-approved protocol to collect PB prior to- and post-TBI conditioning. With approval from the Duke University Institutional Review Board (IRB), between 5–12 mL of peripheral blood was collected from consented patients prior to and 6 hours following TBI with 150 to 200 cGy as part of their pre-transplantation conditioning. Patients undergoing TBI as part of their pre-transplantation conditioning and healthy donors were enrolled to participate in this study following a protocol to collect PB samples that was previously approved by the Duke University Institutional Review Board. All patients receiving non-myeloablative conditioning were treated with 200 cGy of TBI from a linear accelerator at a dose rate of 20 cGy/min. All patients who underwent TBI-based myeloablative allogeneic or autologous stem-cell transplantation received radiation fractionated at 150 cGy per fraction. All patients had PB collected (50 ml) prior to and 6 hours following exposure to either 200 cGy or 150 cGy radiation treatment. A subset of patients also received 30 mg/m^2^ of fludarabine intravenously on days −5 through −2 and 500 mg/m^2^ of cyclophosphamide intravenously on days −5 through −2 as further immunosuppressive therapy. The irradiation was administered one day prior to the initiation of the fludarabine and cyclophosphamide, and the PB samples were drawn prior to the exposure to these immunosuppressive agents.

### Total RNA Isolation

Blood was collected in PAXgene fluid and total RNA was extracted using the RNeasy Protect Animal Blood Kit or Human Blood kit. RNA was beta globin reduced using Ambion Mouse/Rat GLOBINclear kit for the mouse studies and Ambion Human GLOBINclear kit for the human studies. RNA was purified using the Qiagen RNEasy Mini Kit. All total RNA samples were assessed for quality using a NanoDrop ND8000 Spectrophotometer for absorbance ratios, and the Agilent Bioanalyzer 2100 for RIN scores.

### Microarray Data

500 ng of total RNA was amplified according to the MessageAmp Premier protocol (Ambion). Affymetrix GeneChip analysis was performed according to the manufacturer’s instructions, and targets were hybridized to the Mouse 430A 2.0 GeneChip and Human U133A 2.0 GeneChip (Affymetrix, Santa Clara, CA). All microarray data are available on the Gene Expression Omnibus (GEO) database. Mouse data are referenced as GSE 52403 and human data are referenced as GSE 58613.

### CLPA Probe Design and Synthesis

A 10-plex CLPA assay corresponding was developed. The DxDirect probes sets were designed using the “DxDirect Module” of the AlleleID v 7.81 software package from Premier Biosoft (Palo Alto, CA; http://www.premierbiosoft.com/index.html). Each gene was designed to have a unique ligated length that could be differentially detected on a capillary electrophoresis instrument. See **Table 1** for probe sequences and concentrations.

**Table 1:** Sequences of CLPA probes and primers.

Gene	Conc. (pM)	S-Probe (contains 3′ Phosphorothioate modification)
BAX	333.4	5′-GGGTTCCCTAAGGGTTGGACGCGTTCTAAACGGACTGTTACCAGAGTCTGTGTCCACGGCGGCAATCATCCTC-3′
BBC3	1333.4	5′-GGGTTCCCTAAGGGTTGGACGCGTTCTAAATGTACAGAAAATTCATTCCGGTATCTACAGCAGCGCATA-3′
CDKN1A	2666.8	5′-GGGTTCCCTAAGGGTTGGACGCGTCATGCCCTGTCCATAGCCTCTACTGCCACCATC-3′
CDR2	666.7	5′-GGGTTCCCTAAGGGTTGGACGCGGCAACTAAAGATCTCCTTAAACAACGCTTTGTATTCTGGAGG-3′
FDXR	2666.8	5′-GGGTTCCCTAAGGGTTGGACGCGACTCAGTGGAAACAGGCCATTAGACAGATGACCCTCCACAGTCCAGCAGTAGAGAGATGGG-3′
GAPDH	20	5′-GGGTTCCCTAAGGGTTGGACGCGTGGCGAGAGTGTCTCGTATCTCGCTCCTGGAAGATGGTGATGGGATT-3′
MRPS18A	2666.8	5′-GGGTTCCCTAAGGGTTGGACGCGTTCTAAACGGACTGTTACCAGGATGAACTGGCTAAGCAGCAGAACATCGTCA-3′
MRPS5	1333.4	5′-GGGTTCCCTAAGGGTTGGACGGTGCAGTCTTCACATCTTCCCAGTCCAGTTTGACG-3′
MYC	333.4	5′-GGGTTCCCTAAGGGTTGGACGCGTGTTCGGTTGTTGCTGATCTGTCTCAGGACTCTGACAC-3′
PCNA	666.7	5′-GGGTTCCCTAAGGGTTGGACGCGTTCTAAACGGACTGTTACCACTTCACCGCAATTTTATACTCTACAACAAGGGGTACATCTGCAGACA-3′
Gene	Conc. (pM)	SA-Probe (contains 3′ Phosphorothioate modification)
GAPDH	1313.4	5′CGTGGCGAGAGTGTCTCGTATCTCGCTCCTGGAAGATGGTGATGGGATT-3′
Gene	Conc. (pM)	L-Probe (contains 5′ Dabsyl-T modification)
BAX	333.4	5′-TGCAGCTCCATGTTACTGTCCAGTTCGTCCCCACAGGATGAGCCTGCTCTAGATTGGATCTTGCTGGCAC-3′
BBC3	1333.4	5′-TACAGTATCTTACAGGCTGGGCCATCCCTCCCCACAGGATGAGCCTTGGAATGTCGGAAATGCTCTAGATTGGATCTTGCTGGCAC-3′
CDKN1A	2666.8	5′-TTAAAATGTCTGACTCCTTGTTCCGCTGCTAATCTGGCGAGAGGCTCTAGATTGGATCTTGCTGGCAC-3′
CDR2	666.7	5′-TGTTGTAGGGGAACTCACGGGCTCTGGGTTGTTCTAAACGGACTGGCTCTAGATTGGATCTTGCTGGCAC-3′
FDXR	2666.8	5′-TAAGGGGTTAGATCGGCCCACACCTCCACCTTGGCGAGAGCTCTAGATTGGATCTTGCTGGCAC-3′
GAPDH	1333.4	5′-TCCATTGATGACAAGCTTCCCGTTCTCAGCTGGACTCAGTGGAAACAGGCCATTAGACAGAACAGGGCTCTAGATTGGATCTTGCTGGCAC-3′
MRPS18A	2666.8	5′-TAGTTATACTTGTGCTTCAGGTTCCAACGGCAGATGGACAGGATGAGCCTTGGAATGTCGGAAATGCTCTAGATTGGATCTTGCTGGCAC-3′
MRPS5	1333.4	5′-TCTGGAACCTCATCTTCTGGCTCTGGATCCTTCCGCTCTAGATTGGATCTTGCTGGCAC-3′
MYC	333.4	5′-TGTCCAACTTGACCCTCTTGGCAGCAGGATAGTCGCTCTAGATTGGATCTTGCTGGCAC-3′
PCNA	666.7	5′-TACTGAGTGTCACCGTTGAAGAGAGTGGAGTGGCACAGGATGAGCCTTGGAATGTCGGAAATAGGGCTCTAGATTGGATCTTGCTGGCAC-3′
Gene	Conc. (pM)	TC-probe (biotinylated)
MRPS5	2666.8	5′-GGGACGCAACCACAATGGGCAGAGGGC-3′
MRPS5	2666.8	5′-GCGGCTCTCTTCAAATTAGACCACACAGAGCGC-3′
MYC	2666.8	5′-GAGTGGAGGGAGGCGCTGCGTAGTTGTGCT-3′
MYC	2666.8	5′-ATTCTCCTCGGTGTCCGAGGACCTGGGGCTG-3′
CDKN1A	2666.8	5′-GCAATGAACTGAGGAGGGATGAGGTGGATGAGGA-3′
CDKN1A	2666.8	5′-GGAAAGACAACTACTCCCAGCCCCATATGAGCCCA-3′
CDR2	2666.8	5′-GGCCAGTTCCCAGCCGCTGGCAACAGGCTCAGAC-3′
CDR2	2666.8	5′-TGTTCTCTGTTCATCTATTTCCTGCTTAGTTTTC-3′
BAX	2666.8	5′-GCTTGAGACACTCGCTCAGCTTCTTGGTGGAC-3′
BAX	2666.8	5′-GAAAACATGTCAGCTGCCACTCGGAAAAAGACCTCTC-3′
FDXR	2666.8	5′-GGTTACCTCAGTTGCTGAAAGCTAAAACCTTGCGCGAAAAA-3′
FDXR	2666.8	5′-TTTCTTGGTTGCAGCTGTTTTATTTCCAGCATGTTCCCAA-3′
BBC3	2666.8	5′-CAGACTCCTCCCTCTTCCGAGATTTCCCACCCTC-3′
BBC3	2666.8	5′-GGAAACATACAAAAATCATGTACAAAAAAAATTAACC-3′
GAPDH	2666.8	5′-CGGTGCCATGGAATTTGCCATGGGTGGAATCATA-3′
GAPDH	2666.8	5′-GTACTCAGCGCCAGCATCGCCCCACTTGATTTTGG-3′
MRPS18A	2666.8	5′-GGCCAGAGGGGTTAGGAGGATTTGGACTCTCC-3′
MRPS18A	2666.8	5′-CTGTGATCTTTCGGGGCAGCATGCCTCCATG-3′
PCNA	2666.8	5′-TAAAGAAGTTCAGGTACCTCAGTGCAAAAGTTAG-3′
PCNA	2666.8	5′-ATCCTCGATCTTGGGAGCCAAGTAGTATTTTAAGTGTCCC-3′
Primer	Conc. (nM)	PCR Primers
Forward	600	5′-[FAM]GGGTTCCCTAAGGGTTG-3′
Reverse	600	5′-GTGCCAGCAAGATCCAATCT-3′

### CLPA Primers and Dyes

All PCR primers were synthesized by Eurofins Genomics/Operon (Huntsville, AL) and contained a FAM dye label. See **Table 1** for primer sequences.

### CLPA Assay Protocol

All reagents were provided by DxTerity Diagnostics, Rancho Dominguez, CA, and are represented in **Table 2**. Following a previously described method [16], 50 µL of DxCollect stabilized blood, or cultured blood, was mixed in a 96-well plate (Corning Inc., Union City, CA) or a 32-well plates (Axygen Scientific/Corning Inc., Union City, CA) with 15 µL of DirectReact buffer, 15 µL of Mix A containing S-probes, 15 µL of Mix B containing L- and TC-probes and 5 µL of Mix C (a protein digestion/anti-coagulation solution). Four or five technical replicates were performed (as indicated) in all experiments. The plates were sealed with 8-well strip caps (Agilent Technologies, Santa Clara, California) and the reaction mixtures were incubated in a Veriti thermocycler (Life Technologies, Carlsbad, CA) for 5 minutes at 55°C followed by 10 minutes at 80°C and then 2 hours and 45 minutes at 55°C. Next, 5 µL of DirectBeads (streptavidin coated paramagnetic beads) were added to each well and mixed by pipetting. The samples were then incubated for an additional 15 minutes at 55°C to allow ligation complex binding to the beads. The plate was removed from the thermocycler and placed on a 96-well Side Skirted Magnetic Particle Concentrator (Invitrogen, Carlsbad, CA) for 2 minutes to capture the beads to the side of the well. The liquid reaction mixture was aspirated using a multichannel pipette (Rainin, Columbus OH). The beads were washed 3 times with 180 µL DirectWash buffer and the wash buffer was removed. DirectTaq (containing Taq DNA polymerase, complete PCR buffer and dNTPs) and DirectPrime universal primer mix (containing a forward and reverse PCR primer), were then added to the washed beads, and the mixture was amplified by PCR (2 min at 95°C, followed by 30 cycles of 10 s at 95°C; 20 s at 57°C and 20 s at 72°C). The resulting PCR amplified-products (2 µL) were mixed with 17.5 µL Hi-Di Formamide (Life Technologies, Carlsbad, CA), 0.5 µL GeneScan 600 Liz V2 dye Size Standard (Life Technologies) and injected into a Life Technologies 3500×L Dx Genetic Analyzer using the Fragment Analysis Module, according the manufacturers guidelines. The CE instrument was configured with a 24-cap array and POP-6 polymer. Injection time was 15 seconds. The whole process can be completed in less than 6 hours, and is amenable to automation using standard laboratory robotics. The human PB samples for ex vivo analysis via CLPA were collected and processed at the Joint Laboratory of Scottsdale HealthCare, Center for Applied NanoBioscience and Medicine at the University of Arizona, by Dr. Muriel Brengues and Dr. Frederic Zenhausern.

**Table 2:** CLPA Assay Reagents.

Required Reagents	Description
DxCollect BCT	DxCollect Blood Collection Tube. Available from DxTerity Diagnostics
Mix A	Contains S-probes and SA-probe at the concentrations listed in Table 1. Diluent is 1 mM DTT in 1X TE Buffer. Probes are heat activated for 2-min at 95°C after formulation
Mix B	Contains L-probes and TC probes at the concentrations listed in Table 1
Mix C	Protein Digestion and Anti-coagulation solution. Available from DxTerity Diagnostics
DirectReact	CLPA reaction buffer. Available from DxTerity Diagnostics
DirectPrime	2X PCR Primer Mix. Available from DxTerity Diagnostics
DirectTaq	2X PCR Master Mix. Available from DxTerity Diagnostics
DirectBeads	2.7 micron diameter streptavidin coated paramagnetic beads. Available from DxTerity Diagnostics
DirectWash	Wash solution for bead washing steps. Available from DxTerity Diagnostics

### Data Analysis

The capillary electrophoresis data files were processed with GeneMarker Software by SoftGenetics, LLC, version 2.4.0 (State College, PA) in order to generate the peak height RFU values. The peak data tables were saved as txt files and analyzed using JMP v11.0 (SAS Institute, Cary, NC). Gene normalized values were generated by log transforming the raw RFO values obtained from the instrument followed by dividing by the geometric mean of the RFU values of the MRPS5, CDR2 and MRPS18A genes from the same sample.

### CLPA Linear Regression Model

The normalized data for the ex vivo and TBI samples were modelled using the CORExpress statistical analysis software package from Statistical Innovations (Belmont, MA). A correlated component, linear regression model was developed using step-down modelling and 10-fold cross validation. The ex vivo and TBI data sets were modelled together and the dose estimates were outputted from the software package.

### Ortholog Mapping

For determination of mouse–human analogs, we utilized the *Chip Comparer* program (http://chipcomparer.genome.duke.edu) [17]. Annotation mapping resulted in 9150 genes with matching analogs in both mouse and human microarrays.

### Correlation

In light of the potential for outliers, correlation between probe set and radiation exposure was computed using Kendall correlation, a non-parametric test that is more robust than standard Pearson correlation. Correlation was computed between radiation dose and every gene with a human-mouse ortholog. For the mouse and human *ex vivo* samples, those correlations were computed for each exposure time separately as well as for all times taken together. Because dose and time are perfectly confounded in the human TBI samples, in this data set correlation between dose and gene expression was computed only for all of the data.

### Regression Analysis Approach

We are most interested in parsimonious models for estimating radiation exposure from gene expression. In order to obtain short gene lists, we utilized a two-step model building procedure. First, we selected genes with absolute correlation to dose greater than 0.3. Second, those genes were fed into an elastic net regression model to build the predictor. Elastic net is a penalized regression technique which automatically drops out unimportant variables. In order to control for overfitting, we utilized leave-one-out cross-validation. Graphs show results on held out (out-of-bag) samples. Cross-validation was carried out on both steps of the model building.

## Results

### A 15 gene classifier can discriminate radiation dose levels with high accuracy in mice

**Figure 1:**
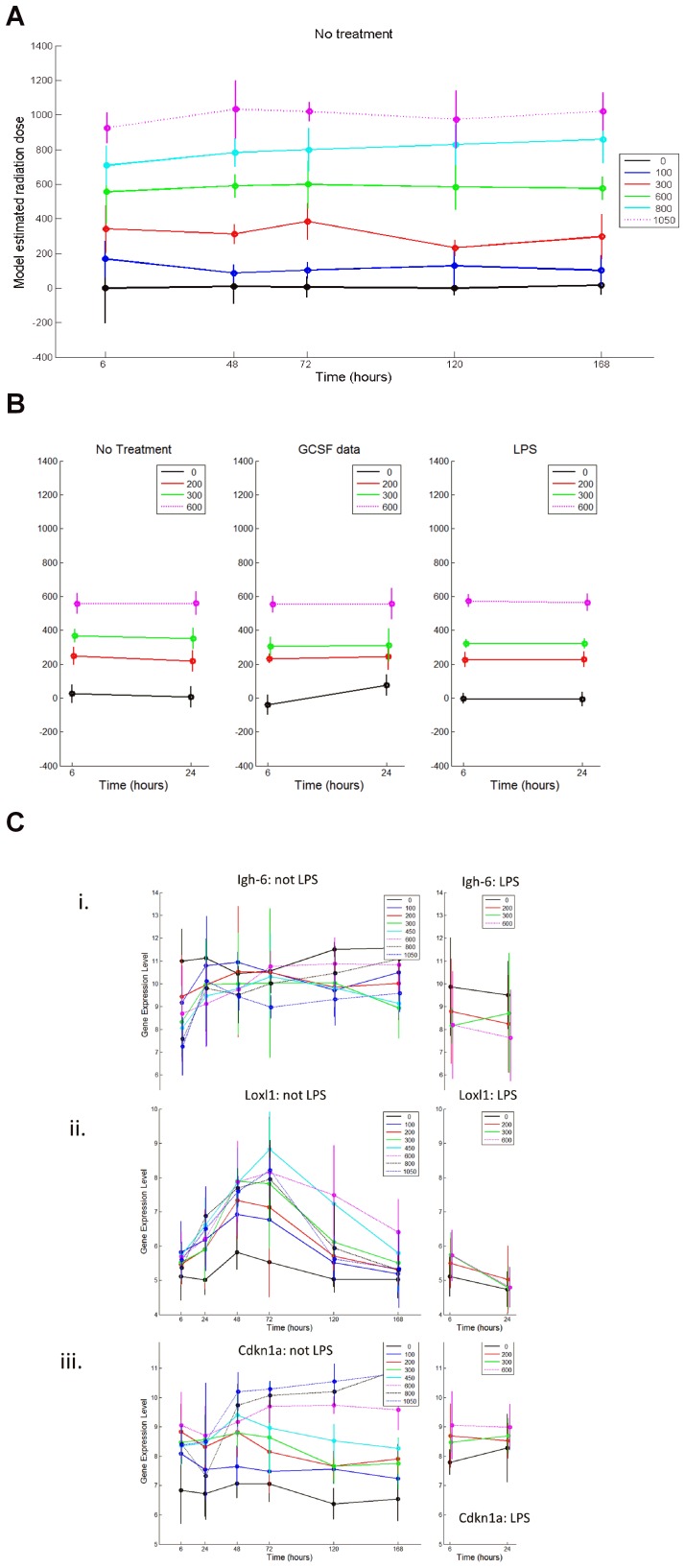
A 15-gene classifier can predict radiation dose levels in mice. A) C57Bl6 mice were irradiated with the TBI dose levels indicated (colors shown) and PB was collected at the times post-irradiation shown on the X axis. The Y axis shows the radiation dose levels predicted by application of the classifier against the irradiated samples. Each dot represents the mean dose level with corresponding 99% confidence intervals about the mean. As shown, at each dose level tested and at every time point through 7 days (168 hrs), the classifier discriminated radiation dose with high accuracy. B) Neither GCSF nor LPS treatments confound the predictive capability of the classifier to predict murine radiation dose levels. The predicted radiation dose levels (y axis) are plotted against time (x axis) of murine PB samples treated with and without GCSF and LPS. C) The RMA normalized gene expression levels of i) IGH-6, ii) LOXL1 and iii) CDKN1A are shown over time following several different radiation dose levels in mice and *ex vivo* with and without LPS treatment. While IGH-6 expression decreased in response to irradiation, LOXL1 expression increased promptly and CDKN1A was a late responsive gene.

We have previously shown that gene expression profiles utilizing 50–100 genes can predict radiation status in mice with a high degree of accuracy [13]–[15]. However, in translating array-based gene expression profiles to a clinically usable assay (e.g. RT-PCR), it will be important to reduce the number of genes within the classifier to the smallest number possible for assay development. We irradiated adult C57Bl6 mice with several medically-relevant doses of total body irradiation (TBI; 0, 100, 300, 600, 800 and 1050 cGy) and examined the gene expression in PB cells at different time points following exposure (6 hrs, 48 hrs, 72 hrs, 96 hrs, 7 days) as well as the gene expression of PB cells from non-irradiated mice (0 hrs). We utilized a variable selection regression approach (described in Methods) to develop a 15-gene gene expression profile which contained genes whose expression strongly correlated with exposure to radiation (**Table 3**). This classifier discriminated radiation dose levels between 0 cGy and 1000 cGy in mice with nearly 100% accuracy (**Figure 1A**). We next examined the predictive capability of this profile in the context of bacterial sepsis and GCSF administration, both of which are expected to potentially confound early analysis of victims in a radiation mass casualty event. The predictor remained highly accurate at discriminating radiation dose levels in mice that were pre-treated with either E. coli-derived lipopolysaccharide (LPS), which mimics bacterial sepsis, or GCSF, which would be administered to many radiation victims (**Figure 1B**). Within the 15-gene classifier, genes could be identified with unique expression responses, including Igh6, an early responsive gene, Loxl1, an intermediate response gene, and CDKN1A, a late response gene (**Figure 1C**). The expression changes in such individual genes provided validation that this 15-gene classifier encompassed genes with particular relevance to the biological response to radiation in the hematopoietic system.

**Table 3:** Murine Radiation Genes.

Affymetrix Probe ID	Gene Symbol	Description
1418648_at	Egln3	EGL nine homolog 3
1421679_s_at	Cdkn1a	Cyclin-dependent kinase inhibitor A
1422303_a_at	Tnfrsf18	Tumor necrosis factor receptor superfamily, member 18
1416295_a_at	IL2rg	Interleukin 2 receptor
1427329_a_at	Ighm	Immunoglobin heavy constant mu
1449025_at	Ifit3	Interferon-induced protein with tetratricopeptide repeats 3
1424042_at	Tmem5	Transmembrane protein 5
1423652_at	Isca1	Iron-sulfur cluster assembly 1 homolog
1427455_x_at	Igkc	Immunoglobulin kappa constant
1425226_x_at	Trbv31	T-cell receptor beta, variable 31
1424828_a_at	Fh1	Fumarate hydratase 1
1451978_at	LoxI1	Lysyl oxidase-like 1
1423345_at	Degs1	Degenerative spermatocyte homolog
1436836_x_at	Cnn3	Calponin 3, acidic
1423182_at	Tnfrsf13b	Tumor necrosis factor receptor superfamily, member 13b

### Classifiers developed in murine models alone have limited accuracy in predicting human radiation status

**Figure 2:**
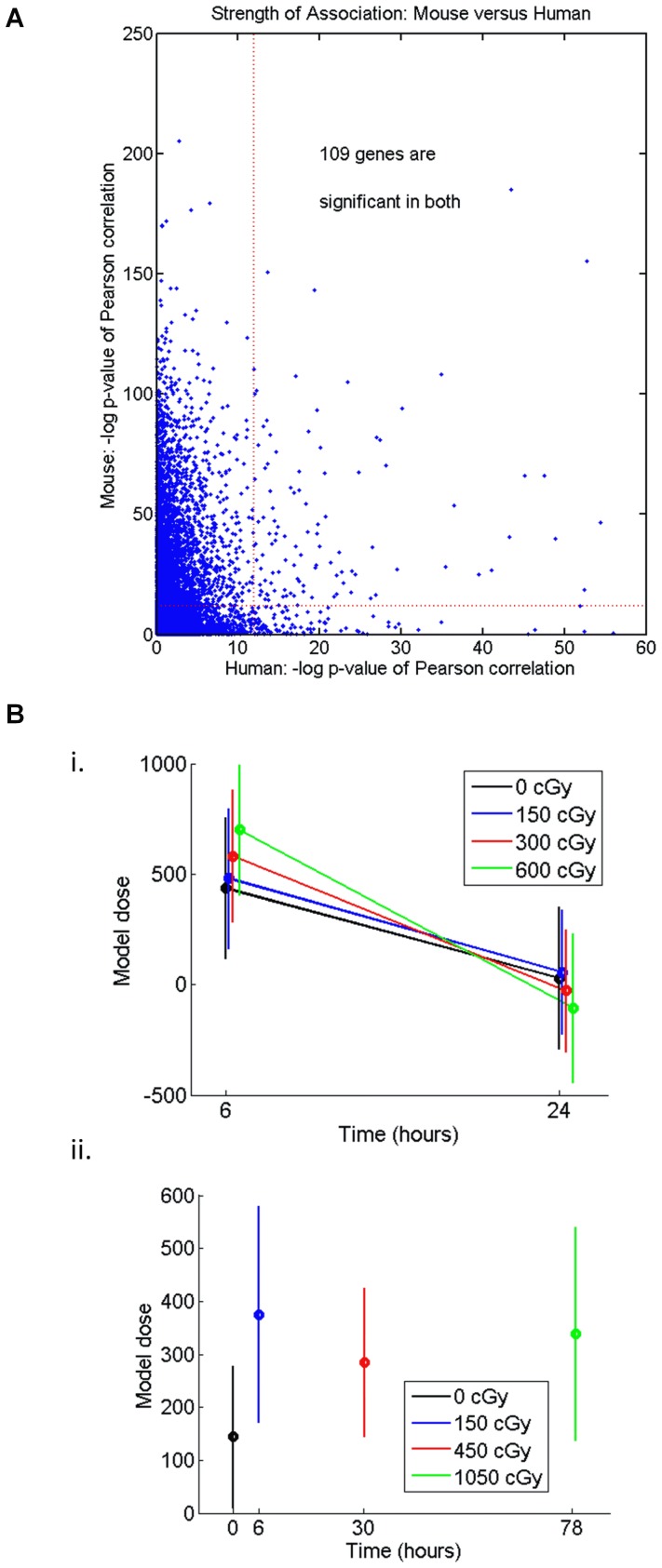
Murine gene expression profiles do not predict human radiation status. A) A Minority of Murine Radiation Response Genes Are Expressed in Humans. The scatterplot shows –log (P-value) for a Pearson test of correlation between radiation exposure dose and gene expression for each gene with a mouse-human analog. If mouse (y-axis) and human (x-axis) were identifying the same genes, then we would expect that the points would fall on a diagonal line. Approximately 9,000 mouse genes have clear human analogues on the U133A microarray. 3,353 genes have significant association with dose (p-value <.05 after correction for multiple testing). Of those, 109 genes are significantly associated in humans. B) Performance of the Murine Radiation Classifier Against Human PB Samples. We used penalized regression with individual genes as potential independent variables to build a model predicting radiation exposure dose in mice. However, using variable selection to build models that predict radiation in mice does not lead to models that predict radiation exposure in humans. *Panel i* shows the mean predicted radiation levels (+/− SEM) of human *ex vivo* irradiated blood samples at 6 hrs and 24 hrs. *Panel ii* shows the mean predicted radiation levels of human TBI patient samples. The x-axis shows the times after irradiation at which gene expression measurements were taken. Actual radiation doses are displayed by different colors. Note the overlap between predicted mean radiation values in each analysis.

Prior studies from our laboratory and others have suggested that genes contained within a murine PB molecular profile of radiation injury might be useful in predicting the radiation status of humans [13], [18]. Particular genes, such as DDB2 and CDKN1A, have also been suggested to be potentially useful in predicting radiation status in mice and humans [14]. However, no study has formally tested whether gene expression profiles of radiation response generated from mice samples could predict the radiation status of humans. Here, we tested whether the murine signature which we found to be highly predictive of radiation status in mice could predict the radiation status and discriminate radiation dose levels in human PB samples (**Figure 2**). From our list of mouse-human orthologs, we found 3,353 genes which had a significant correlation in expression with radiation dose in mice (**Figure 2A**). Of these, only 109 genes (3%) were found to be significantly associated with radiation dose in humans. Furthermore, we observed a lack of correlation between the direction of the responses of genes in mice and humans to irradiation (data not shown). Moreover, when we tested the murine predictor of radiation injury (15 genes, **Table 3**) against human PB samples, it poorly predicted the radiation status of either human ex vivo irradiated PB samples or PB samples from human TBI patients (**Figure 2B**). These results suggest that molecular signatures generated in mice models alone are not useful for development of a gene expression assay for human radiation injury.

### A human radiation classifier predicts radiation status and discriminates dose levels in human cells

**Figure 3:**
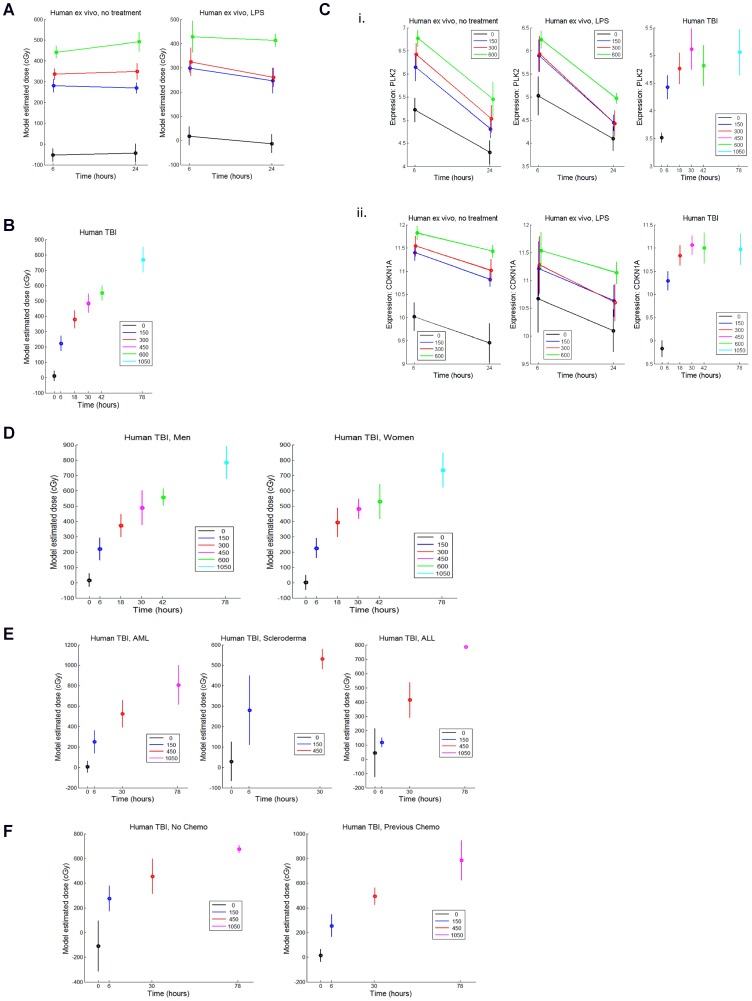
A radiation classifier generated from human genes predicts human radiation exposure with high accuracy. A) At left, the mean +/− SEM estimated radiation doses of human PB cells are plotted on the y axis at 6 hrs and 24 hrs of culture (x axis) following ex vivo irradiation. At right, identical radiation dose prediction is shown in the presence of LPS. Green line: 600 cGy, Red line; 300 cGy, Blue line: 150 cGy, Black line: 0 cGy. B) The mean +/− SEM estimated radiation dose levels of human PB from TBI patients are plotted on the y axis. The x axis shows the time points after initial TBI dose at which the PB sample was collected from the patient. PB was collected at 6 hours following the first fraction of TBI (150 cGy) and 12 hours later (18 hr time point, following the 2^nd^ fraction = total 300 cGy) and so on. TBI was delivered in a standard fractionated manner to patients, twice daily (150 cGy separated by 6 hours) and so these time points were fixed by the clinical treatment protocol. The legend shows the actual cumulative radiation dose received at each time point. C) The responses of individual human genes, PLK2 and CDKN1A, are shown in response to ex vivo irradiation and TBI. *Panel i*. Expression of PLK2 is shown on the y axis at 6 hours and 24 hours after ex vivo irradiation, with and without LPS (left); at right, the expression of PLK2 is shown in the PB samples of TBI patient samples at the radiation dose levels shown. *Panel ii.* Expression of CDKN1A is shown in ex vivo irradiated human PB samples, with and without LPS (left); at right, the expression of CDKN1A is shown in human TBI patient samples at different radiation dose levels. D) The model estimated radiation dose levels for the human TBI patient samples are shown on the y axis, separated by gender. The actual radiation dose levels are indicated in the legend. The x axis shows the time points at which PB samples were collected. E) The model estimated radiation dose levels for the human TBI patient samples are shown on the y axis, separated by the diagnoses, acute myeloid leukemia (AML), scleroderma, and acute lymphocytic leukemia (ALL). Note that for some diagnostic groups, not all radiation dose levels are represented. F) The model estimated radiation dose levels for human TBI patient samples are shown on the y axis, separated by “no chemotherapy” or “previous chemotherapy.”

In light of the poor predictive capability of a murine gene signature toward predicting human radiation status, we sought instead to develop a human radiation predictor utilizing human *ex vivo* PB samples and human TBI patient samples. In order to develop a human gene expression profile of radiation response, we collected PB from healthy donors (n = 7 males, 8 females, ages 44–61) and measured the global gene expression after *ex vivo* with irradiation with 150 cGy, 300 cGy, or 600 cGy. To complement this, we also measured gene expression in PB cells from patients who received TBI as part of their conditioning prior to autologous or allogeneic stem cell transplantation (n = 45 patients, 22 females, 23 males, ages 21–66, **Table S1**). We utilized our variable selection regression approach, coupled with a list of genes which we have previously described [13], [14] and a subset from the literature [18], to generate a predictor of human radiation exposure. We utilized variable selection regression with interaction effects for time and treatment to refine the list of potential predictors further. This approach revealed a clear signature of gene expression which was associated with human irradiation and an optimized group of 18 genes (**Table 4**). Our optimized human radiation classifier distinguished irradiated from non-irradiated human PB cells with 100% accuracy (**Figure 3A**). Importantly, when human PB samples which had been irradiated ex vivo with different, medically-relevant doses of radiation were tested (150 cGy, 300 cGy, 600 cGy), this human classifier was able to predict radiation dose levels with high accuracy (**Figure 3A**). We also found that exposure to LPS did not significantly reduce the accuracy of the human classifier in predicting the radiation status of human *ex vivo* irradiated PB cells (**Figure 3A**). To further test the capability of the human classifier to predict radiation dose levels in a clinically relevant population, we tested the capacity of our 18 gene classifier to distinguish radiation dose in adult patients who were irradiated with several different dose levels of TBI (150 cGy, 300 cGy, 450 cGy, 600 cGy and 1050 cGy; **Figure 3B**). The human classifier was able to discriminate the different irradiated human populations as well as non-irradiated healthy adults with high accuracy. Of note, strong discrimination was achieved between 0 cGy (non-irradiated) and 150 cGy, a dose level above which medical interventions (e.g. administration of GCSF or antibiotics) would be utilized. Accurate discrimination of non-irradiated from irradiated humans in this dose range will be critical to allow separation of the “worried well” from those who have suffered true radiation injury in a mass casualty event. Additionally, the human classifier accurately discriminated those irradiated with 300 cGy versus 450 cGy, dose levels which bracket a medical “tipping point” between a sub-lethal exposure and an exposure level (450 cGy) which would require major medical interventions to avoid life-threatening complications (e.g. hematopoietic failure)(**Figure 3B**) [2], [3]. Of note, evaluation of individual gene response revealed several genes, including PLK-2 and CDKN1A, which displayed unique amplitude and kinetics of expression response in response to TBI (**Figure 3C**). The expression of PLK-2 and CDKN1A, which are both involved in cell cycle regulation and cellular repair processes [19], [20], provided biological validation of the genes selected for this human radiation classifier.

**Table 4:** Human Radiation Genes.

Affymetrix Probe ID	Gene Symbol	Gene Description
201939_at	PLK2	polo-like kinase 2
201202_at	PCNA	proliferating cell nuclear antigen
203409_at	DDB2	damage-specific DNA binding protein 2, 48kDa
202284_s_at	CDKN1A	cyclin-dependent kinase inhibitor 1A (p21, Cip1)
200974_at	ACTA2	actin, alpha 2, smooth muscle, aorta
201863_at	FAM32A	family with sequence similarity 32, member A
202431_s_at	MYC	v-myc myelocytomatosis viral oncogene homolog
201839_s_at	EPCAM	epithelial cell adhesion molecule
200642_at	SOD1	superoxide dismutase 1, soluble
202079_s_at	TRAK1	trafficking protein, kinesin binding 1
203065_s_at	CAV1	caveolin 1, caveolae protein, 22 kDa
201338_x_at	GTF3A	general transcription factor IIIA
202119_s_at	CPNE3	copine III
202786_at	STK39	serine threonine kinase 39
203048_s_at	TTC37	tetratricopeptide repeat domain 37
202991_at	STARD3	StAR-related lipid transfer (START) domain
202081_at	IER2	immediate early response 2
201092_at	RBBP7	epithelial cell adhesion molecule

We next sought to determine if the capability of the human radiation classifier toward discriminating radiation dose levels in humans could be confounded by particular variables, such as gender, underlying diagnosis or prior chemotherapy exposure. Importantly, gender had no impact on the accuracy of the human radiation predictor (**Figure 3D**). We also observed no substantial impact of underlying diagnosis or prior chemotherapy exposure on the predictive accuracy of the human radiation classifier (**Figure 3E, F**).

### A CLPA-based assay can predict radiation status and discriminate radiation dose levels of human blood

**Figure 4:**
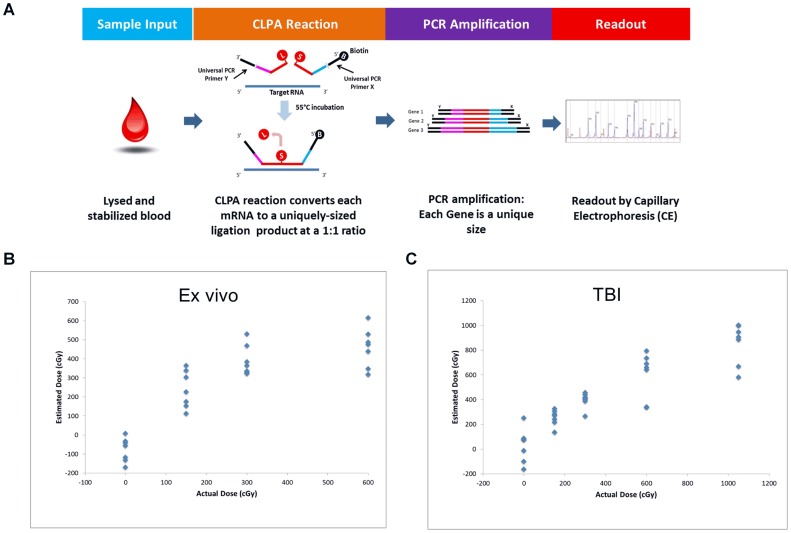
The CLPA assay predicts the radiation status of human PB samples with high accuracy. A) A schematic representation of the CLPA assay is shown. B) Scatter plots are shown of the predicted radiation dose levels of human PB samples (y axis) which were irradiated *ex vivo* with the radiation dose levels shown on the x axis. C) Scatter plots are shown of the predicted radiation dose levels of PB samples from human TBI patients (y axis) who were irradiated with the dose levels shown on the x axis. n = 7 patient samples in each group.

The studies above demonstrate the power of utilizing genome-wide analysis of gene expression in a radiosensitive cell population (PB cells), utilizing multiple model systems, and coupled with sophisticated computational tools, to develop a classifier capable of predicting human radiation status and radiation dose level in humans with a high degree of accuracy. However, translation of such a classifier for application in a mass casualty scenario would require the development of an assay which is more automated, capable of higher throughput and provides more rapid results than research-level gene expression arrays (e.g. Affymetrix), while hopefully delivering a comparable level of radiation dose discrimination. To meet this objective, we have incorporated several genes from our human radiation classifier, coupled with additional genes (**Table 5**), into a chemical ligation-dependent probe amplification (CLPA) assay developed by DxTerity Diagnostics. CLPA requires no isolation of RNA or cDNA synthesis, but rather, CLPA uses a non-enzymatic chemical ligation reaction to produce synthetic, single stranded DNA fragments in a 1∶1 ratio to the input RNA material. The resulting DNA fragments are isolated by magnetic bead capture, amplified by PCR using common primer sequences, and then assayed using capillary electrophoresis (CE) (**Figure 4A**).

**Table 5:** CLPA Assay Genes.

Gene Name	Gene Symbol	Ref Seq ID	Ligated Product Length (bp)
Mitochondrial ribosomal protein S5	MRPS5	NM_031902	115
v-myc avian myelocytomatosis viral oncogene homolog	MYC	NM_002467	120
Cyclin-dependent kinase inhibitor 1A	CDKN1A	NM_000389	125
Cerebellar degeneration-related protein 2	CDR2	NM_001802	135
BCL2-associated X protein	BAX	NM_138761	141
Ferredoxin reductase	FDXR	NM_024417	148
BCL2 binding component 3	BBC3	NM_001127240	155
Glyceraldehyde-3-phosphate dehydrogenase	GAPDH	NM_002046	161
Mitochondrial ribosomal protein S18A	MRPS18A	NM_018135	165
Proliferating cell nuclear antigen	PCNA	NM_002592	180

The CLPA assay was used to test *ex vivo* irradiated blood samples (0, 150 cGy, 300 cGy, and 600 cGy) from 7 healthy donors and Human TBI blood samples (0, 150 cGy, 300 cGy, 600 cGy and 1050 cGy) from 7 cancer patients. A linear regression based model was developed using the CORExpress data analysis software package from Statistical Innovations (Belmont, MA). The radiation dose estimates produced by CLPA assay of human blood samples that were irradiated *ex vivo* are shown in **Figure 4B** and the dose estimates of samples from the human TBI patients are shown in **Figure 4C**. Importantly, the same model was able to estimate dose for both sample sets with comparable accuracy.

## Discussion

The threat of terrorism via detonation of radiological weapons or improvised nuclear devices remains a major public health and national security concern in the United States [1]–[4]. While federal, state and private institutions have marshaled resources and expertise to prepare a medical response for a radiological mass casualty event, a major gap remains in the tools available to health care providers for biodosimetry [4]–[12]. In order to address this gap in knowledge and medical triage capability, we have performed research to determine if PB gene expression profiles which are associated with radiation response in mice and humans could be utilized to predict radiation status and discriminate medically relevant levels of radiation exposure [13]–[15]. The results of these studies have provided proof of principle that the gene expression profile of a subset of genes could be applied to predict radiation status and discriminate dose level with a high degree of accuracy. However, important questions remained. For example, what was the limiting number of genes necessary for the profile to remain highly accurate and predictive over a range of radiation dose levels and time points following exposure? Do the genes that predict radiation status in mice also predict human radiation status or would a predictor built solely via analysis of human blood samples provide the most accurate human biodosimetric assay? Lastly, could a translatable assay for radiation exposure be developed which is more rapid, high throughput and as accurate as an array-based assay? In this report, we demonstrate that a set of as few as 18 genes can be utilized to predict human radiation status with >90% accuracy and durable accuracy over 7 days post-TBI. Going forward, we will be performing additional experiments to refine the human radiation classifier and perhaps decrease the number of essential genes further. It remains possible that a smaller subset of genes could be utilized at a single time point to predict radiation status and perhaps even discriminate a single dose level from all others in a particular time window.

Mice models represent attractive tools to study the biological response of various organ systems to ionizing radiation. Recent studies in mice have suggested that alterations in the expression of particular genes, or particular gene expression profiles may serve as biomarkers of radiation injury and have the potential to be utilized to predict human radiation status [13]–[15], [18]–[25]. Here, we directly tested whether a gene expression profile that was developed in mice and showed high accuracy of prediction of radiation status in mice could also predict human radiation status. Interestingly, the murine radiation predictor demonstrated poor accuracy in predicting human radiation status when tested against *ex-vivo* irradiated human PB or PB samples from TBI patients. At the most basic level, only a small percentage of genes within murine PB demonstrated comparable alterations in gene expression as observed in human PB cells in response to the same dose of radiation. Furthermore, we have found that individual gene responses to radiation were frequently in opposition between mice and humans. This may be explained, at least in part, by the innately higher frequency of PB lymphocytes in mice versus humans, which would yield a different molecular response compared to human PB, which has a higher frequency of neutrophils [26]. Nonetheless, our results suggest that the inclusion of murine genes in the generation of a molecular predictor of human radiation status would be problematic and likely inferior to a predictor built from analysis of human samples.

In light of the inaccuracy of murine radiation genes in predicting the status of irradiated human PB samples, we developed a predictor of human radiation status utilizing solely genes identified from analysis of human ex vivo irradiated PB samples and human TBI samples. This 18 gene classifier was highly accurate in predicting the radiation status and discriminating radiation dose levels for ex vivo irradiated human samples and for human TBI patients. Importantly, the accuracy of this predictor was not confounded by concomitant exposure to LPS (ex vivo) and was not impacted by gender. We also did not observe a substantial impact of the underlying diagnosis or the most recent chemotherapy regimens received by the TBI patients on the capability of this classifier to discriminate radiation dose levels in TBI patients. Of note, the sample size in this study was relatively small and numerous diagnoses and chemotherapy regimens were represented within the cohort; therefore, additional patient samples would be required to confirm whether specific diagnoses or chemotherapy regimens could alter the accuracy of this human radiation predictor. In addition, human TBI patients in this study were irradiated at a dose rate of 20 cGy/min, whereas human ex vivo irradiated samples were irradiated at a dose rate of 480 cGy/min, and it is possible that this difference in dose rate contributed to molecular changes observed in the ex vivo PB samples compared to the human TBI patient samples [27].

An important additional consideration when developing a gene expression-based predictor is the recognition of the impact that the analytical method can have upon the genes which are identified to be important [28]. This variable was highlighted in prior studies of the effects of radiation on gene expression in human PB [28]–[30], in which different methods were utilized to identify clusters of genes which were radiation responsive in each model [28]–[30]. Here, we utilized an elastic net regression model for the specific purpose of maximally reducing our gene list for application in a point-of-care assay system which would allow only a limited number of genes to be represented. We noted little overlap between our human radiation classifier and the gene clusters identified in the prior analyses [28]–[30]. In this regard, our study confirms that the analytical method utilized can substantially impact the genes which are represented in a given radiation classifier.

The ultimate objective for any molecular profile of radiation injury would be the translation and development of such an assay to facilitate the diagnosis and management of radiation injury in people. In order for a molecular assay for radiation injury to be useful for a mass casualty scenario, it must be high throughput, automated, provide rapid sample-to-assay results, be user-friendly and have high accuracy. Here, we describe a novel methodology, CLPA, to predict the radiation status of human PB samples. CLPA provides sample-to-assay results within 6 hours, allows for high throughput and, in preliminary studies, discriminated medically relevant radiation dose levels in human PB samples. It is important to emphasize that substantial additional testing of human clinical samples will be necessary to confirm the capability of CLPA as a diagnostic assay for acute radiation exposure. Nonetheless, our results illustrate the potential for translation of a genetic signature of acute radiation exposure into a candidate, practical biodosimetric assay.

## Supporting Information

Table S1 **The clinical characteristics of the patients whose peripheral blood samples were analyzed are shown.**
(PDF)
