# Supplementary material for: A Translatable Predictor of Human Radiation Exposure
Source: PLoS One. 2014 Sep 25;9(9):e107897. doi: 10.1371/journal.pone.0107897 (PMC4177872; doi:10.1371/journal.pone.0107897)
Supplement: Table S1 — The clinical characteristics of the patients whose peripheral blood samples were analyzed are shown. (PDF) [file pone.0107897.s001.pdf]

Table S1. Patient Characteristics

Characteristic	Number
Patients	n=45 patients (22 females, 23 males)
Patient Age	21 – 66 years (range)
Diagnoses	AML/MDS (n=23)<br>ALL (n=8)<br>Scleroderma (7)<br>Non-Hodgkin's Lymphoma (n=5)<br>CML (n=1)<br>CLL (n=1)
Prior chemotherapy	n=37

Patients undergoing either non-myeloablative or myeloablative allogeneic or autologous stem cell transplantation were eligible for enrollment. All irradiation conditioning was completed 18 hours prior to the initiation of additional immunosuppressive conditioning drugs. PB samples were collected prior to and 6 hours following 200 cGy total body irradiation (non-myeloablative conditioning) or prior to and 6 hours following the first fraction (150 cGy) of total body irradiation (myeloablative conditioning). MDS=myelodysplastic syndrome, AML=acute myelogenous leukemia, ALL=acute lymphocytic leukemia, CML=chronic myelogenous leukemia, CLL=chronic lymphocytic leukemia
